# A longitudinal study of pupillary light reflex in 6- to 24-month children

**DOI:** 10.1038/s41598-020-58254-6

**Published:** 2020-01-27

**Authors:** Clare Kercher, Leila Azinfar, Dinalankara M. R. Dinalankara, T. Nicole Takahashi, Judith H. Miles, Gang Yao

**Affiliations:** 1 Department of Biomedical, Biological & Chemical Engineering, University of Missouri, Columbia, MO 65211 USA; 2 Department of Computer Engineering, University of Sri Jayewardenepura, Nugegoda, Sri Lanka; 3 Thompson Center for Autism and Neurodevelopmental Disorders, University of Missouri, Columbia, MO 65211 USA

**Keywords:** Biomarkers, Paediatric research, Translational research, Autism spectrum disorders

## Abstract

Pupillary light reflex (PLR) is an involuntary response where the pupil size changes with luminance. Studies have shown that PLR response was altered in children with autism spectrum disorders (ASDs) and other neurological disorders. However, PLR in infants and toddlers is still understudied. We conducted a longitudinal study to investigate PLR in children of 6–24 months using a remote pupillography device. The participants are categorized into two groups. The ‘high risk’ (HR) group includes children with one or more siblings diagnosed with ASDs; whereas the ‘low risk’ (LR) group includes children without an ASD diagnosis in the family history. The participants’ PLR was measured every six months until the age of 24 months. The results indicated a significant age effect in multiple PLR parameters including resting pupil radius, minimal pupil radius, relative constriction, latency, and response time. In addition, the HR group had a significantly larger resting and minimal pupil size than the LR group. The experimental data acquired in this study revealed not only general age-related PLR changes in infants and toddlers, but also different PLRs in children with a higher risk of ASD.

## Introduction

Autism Spectrum Disorders (ASDs) are complicated disorders that are marked by persistent deficits in social communication and interactions and by restricted, repetitive patterns of behavior, interests or activities^1^. Initially chronicled 75 years ago^2^, ASDs now affect about 2.47% children and adolescents in USA alone^3^. Although the etiology of ASD is still not fully understood, our understanding of this disorder has since been significantly improved owing to a large amount of physiological, psychological, and neurological studies. Evidence supports that the outcome in children with ASDs can be greatly improved by using early behavioral intervention^4,5^. Unfortunately, most children do not receive an ASD diagnosis until after the age of four^6^, although early signs may appear as young as 12 months of age^7^. Therefore, there is a great interest in finding effective biological markers for early screening of risk of autism and assessing responses to interventions.

Pupillary light reflex (PLR) is the involuntary and nearly instantaneous pupil size change that occurs as a response to the luminous intensity of light that falls on the retina. The pupil size is controlled by the dilator and sphincter muscles innervated primarily by the sympathetic and parasympathetic branches of the autonomic nervous system (ANS), respectively^8^. In 1961, Rubin observed that the pupils in 7 to 12 years old children with ASD constricted slower in responses to light adaption compared to typically developing children^9^. Using a computerized pupillography system, Fan *et al*. discovered that pupils of children with ASD took a greater amount of time to respond to short (0.1 s) light stimuli and constricted less and more slowly than those with typical development^10^. Similar atypical PLR responses were also reported in subsequent studies in children with ASD of different ages using pupillography and eye-tracking devices^11–13^. In addition, studies have shown that quantitative PLR responses were associated with sensory behaviors and autism traits^14,15^. The PLR’s potential for early identification of risk of autism was recently demonstrated by Nyström *et al*.^16^. They reported that the pupil constricted more in 9- to 10-month old infants who later received an ASD diagnosis and the amount of PLR constriction was correlated with the severity of ASD symptoms.

Resting pupil size and PLR parameters are known to change with age. Existing literature indicates that resting pupil size increases from infants to teenagers^17–19^ and then decreases with age thereafter^20–22^. In comparison with resting or static pupil size, there are limited studies on age effect on PLR. Still, current evidence indicates that PLR parameters can change with age^19^. Studies suggested that the age trend might be altered in association with ASD. For example, PLR latency (the delay between stimulation onset and the beginning of pupil constriction) decreased from 6 to 8 years in children of typical development; this trend was not apparent in age-matched children with ASD^11^, suggesting that the PLR differences between individuals with and without ASD may change with age. Interestingly, Nyström *et al*. later reported that the PLR latency was shorter in young children with high-risk of ASD^23^, in contrast with reports that older children with ASD had longer latency than typically developing children^10,11^. Exiting experimental evidence^12^ suggested that different age trends may explain apparent inconsistencies in ASD associated atypical resting pupil sizes reported in the literature^24–26^.

Despite the importance of age effect on PLR, no age-dependent longitudinal study was reported in literature. In particular, age-dependent PLR data in infants and toddlers are scarce due to the challenges in measuring PLR in young children. This study used the recently developed remote PLR (rPLR) system to investigate the PLR changes in children from 6 to 24 months old. This novel rPLR system is capable of imaging pupil size changes at a high spatial resolution without the need of any restrain during the test, which makes it ideal to test PLR in young children^27^. The participants were categorized into two groups based on their susceptibility to ASD. The risk of younger siblings developing an ASD is significantly higher if an older sibling has an ASD diagnosis^28^. Therefore, the ‘high risk’ (HR) group includes children with one or more siblings diagnosed with ASD. On the other hand, the ‘low risk’ (LR) group includes children not associated with ASD in the family history. We intended to answer the following questions: (1) whether the PLR parameters are age-dependent in the 6–24 months of age range, and (2) whether any atypical parameters exist in the ‘high risk’ group of children.

## Results

**Figure 1:**
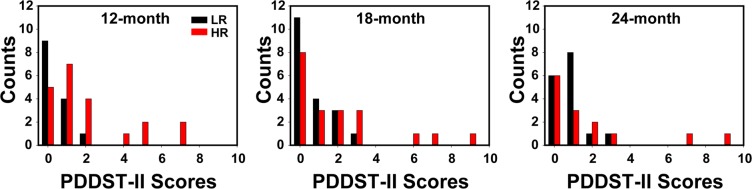
Distribution of the PDDST-II scores obtained at 12-, 18-, and 24-month in both LR and HR groups.

The Pervasive Developmental Disorders Screening Test-II (PDDST-II) scores were recorded as a simple screening for neurodevelopmental disorders in the participants from 12- to 24-month old. Figure 1 shows the distribution of the PDDST-II scores at 12-, 18-, and 24-month. In the HR group, the PDDST-II score changed from 2.05 ± 2.22 at 12-month, to 2.00 ± 2.60 at 18-month, and 1.86 ± 2.80 at 24-month. A few children (4 at 12-month, 3 at 18-month, 2 at 24-month) in the HR group had a score of 5 or above, suggesting potential developmental disorders^29^. In the LR group, the PDDST-II score appeared to increase slightly with age from 0.43 ± 0.65 at 12-month, to 0.68 ± 0.95 at 18-month, and 0.81 ± 0.83 at 24-month. However, none of the participants in the LR group scored more than three.

**Figure 2:**
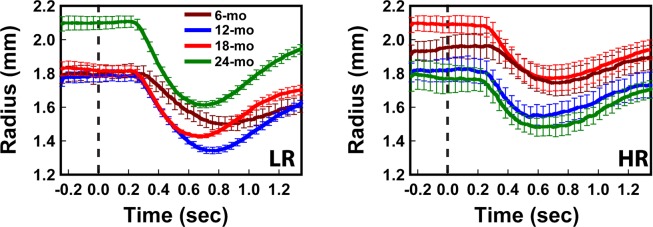
Example mean PLR curves obtained from a subject in the LR group and a subject in the HR group at different ages. The errors bars indicate the standard error. The dashed lines indicated the time of the stimulation flashes.

Figure 2 illustrates example pupilograms obtained from two participants in the LR and HR groups who completed all 4 tests at different ages from 6-month to 24-month. The pupilogram curves shown were averaged results from all successful trials obtained during a single PLR test. The PLR followed the typical PLR curve as those observed in the older children. The pupil size was relatively stable before the stimulation (marked as dashed lines). The pupil then started to constrict after a delay (the latency period), reached a minimum, and then started to recover back to the baseline. Overall, the amount of constriction in the HR group appeared to be slightly smaller than the LR constriction.

**Figure 3:**
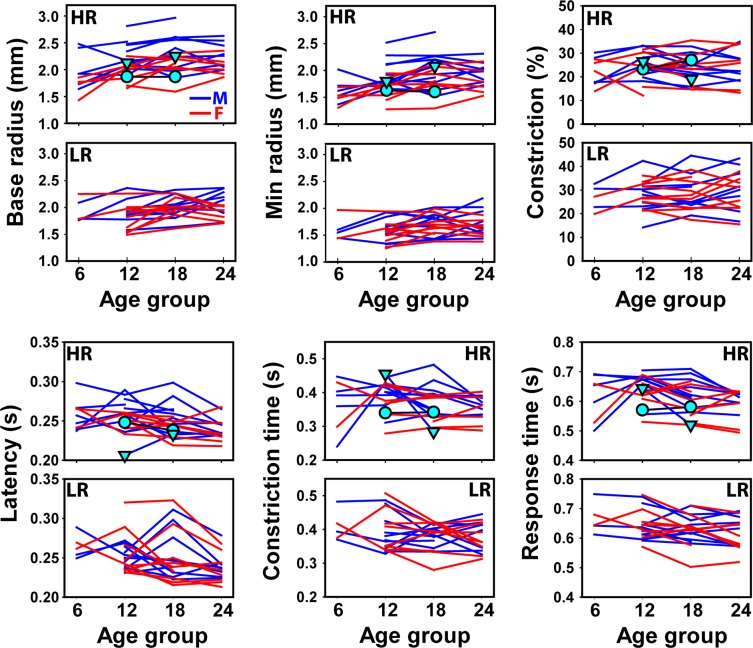
The extracted PLR parameters (base radius, minimal radius, relative constriction, latency, constriction time, and response time) in all participants at different age groups. The data were separated into the high-risk (HR) and low-risk (LR) groups and males (M) and females (F) in each group. The symbols indicated data from the two HR participants who received diagnoses at the end of this study with the triangles representing the one diagnosed with an ASD and the circles representing the other with a diagnosis of global developmental delay.

Figure 3 shows all extracted PLR parameters at different ages in both the HR and LR groups (see also Supplementary Fig. S1). As a group, the base pupil radius, minimal pupil radius, and relative constriction all increased with age; whereas the latency, response time, and constriction time showed a decreasing trend with age. However, there were considerable variations among subjects, which justified the use of a random intercept in the linear mixed-effects model (LMM) analysis. In addition, the HR group appeared to have a larger pupil than the LR group. Both the base and minimal pupil radii appeared to be larger in the males. No clear group or sex effect was observed in the other four parameters. The data from the two HR participants who received diagnoses at the end of this study were labeled using symbols in Fig. 3. The triangle symbol represented the one with an ASD diagnosis and the circle represented the other with a diagnosis of global developmental delay.

The above observations were examined using the LMM analysis. Table 1 shows the estimations of fixed effects and the corresponding 95% confidence intervals (CI). The group (HR vs LR) had a significant effect in three PLR parameters: base radius (F = 10.02, p = 0.003), minimal radius (F = 11.62, p = 0.001), and relative constriction (F = 5.82, p = 0.020). In comparison with the LR group, the pupils in the HR group were bigger before stimulation (t = 3.17, p = 0.003), remained bigger at the maximal constriction (t = 3.41, p = 0.001); but the relative constriction was smaller (t = −2.41, p = 0.020). All timing parameters (latency, response time, and constriction time) were similar in the HR and LR groups.

**Table 1:** Estimates of fixed effects obtained using the linear mixed-effects model (LMM).

	Parameter	Estimate	Std. Error	df	t	Sig.	95% CI [Lower, Upper]
Based radius (mm)	Intercept	2.11	0.06	53.05	33.74	0	[1.98, 2.23]
	[Group = HR]^a^	0.21	0.07	41.36	3.17	0.003	[0.08, 0.35]
	[Sex = F]^b^	−0.19	0.07	41.30	−2.90	0.006	[−0.33, −0.06]
	[Age = 6]^c^	−0.26	0.05	90.54	−5.46	0	[−0.36, −0.17]
	[Age = 12]	−0.13	0.03	84.50	−3.75	0	[−0.20, −0.06]
	[Age = 18]	−0.02	0.03	83.80	−0.46	0.649	[−0.08, 0.05]
Min radius (mm)	Intercept	1.77	0.06	50.26	27.90	0	[1.65, 1.90]
	[Group = HR]^a^	0.23	0.07	40.48	3.41	0.001	[0.10, 0.37]
	[Sex = F]^b^	−0.17	0.07	40.52	−2.45	0.019	[−0.31, −0.03]
	[Age = 6]^c^	−0.21	0.05	87.43	−4.48	0	[−0.30, −0.12]
	[Age = 12]^c^	−0.09	0.03	82.66	−2.90	0.005	[−0.16, −0.03]
	[Age = 18]^c^	0.01	0.03	81.84	0.18	0.858	[−0.06, 0.07]
Constriction (%)	Intercept	29.32	1.69	51.43	17.40	0.000	[25.94, 32.70]
	[Group = HR]^a^	−4.38	1.81	41.40	−2.41	0.020	[−8.04, −0.71]
	[Age = 6]^c^	−4.10	1.24	88.39	−3.32	0.001	[−6.55, −1.64]
	[Age = 12]^c^	−0.91	0.87	83.61	−1.05	0.299	[−2.65, 0.82]
	[Age = 18]^c^	−1.12	0.87	82.80	−1.30	0.199	[−2.85, 0.60]
Latency (ms)	Intercept	240.62	5.17	66.50	46.51	0	[230.29, 250.95]
	[Age = 6]^c^	20.68	5.36	99.11	3.86	0	[10.05, 31.31]
	[Age = 12]^c^	15.59	3.92	86.23	3.97	0	[7.79, 23.39]
	[Age = 18]^c^	11.51	3.90	85.36	2.95	0.004	[3.75, 19.27]
Response time (ms)	Intercept	618.36	13.36	56.24	46.30	0	[591.60, 645.11]
	[Age = 6]^c^	28.06	12.09	89.35	2.32	0.023	[4.03, 52.09]
	[Age = 12]^c^	36.28	8.74	80.33	4.15	0	[18.89, 53.66]
	[Age = 18]^c^	13.58	8.60	79.33	1.58	0.118	[−3.53, 30.68]

^a^The LMM model used results from LR group as the reference.

^b^The LMM model used results from the male group as the reference.

^c^The LMM model used results from the 24-month group as the reference.

The LMM analysis revealed that the sex effect was significant only in the two pupil-size related parameters: base radius (F = 8.41, p = 0.006) and minimal radius (F = 6.01, p = 0.019). As shown in Table 1, in comparison with the boys, the girls had smaller based pupil radius (t = −2.90, p = 0.006) and minimal pupil radius (t = −2.45, p = 0.019).

The LMM analysis indicated that age had a significant effect on base radius (F = 14.00, p < 0.001), minimal radius (F = 10.52, p < 0.001), relative constriction (F = 3.74, p = 0.014), latency (F = 7.13, p < 0.001), and response time (F = 6.34, p = 0.001). In constriction time, the LMM showed a marginal age effect (F = 2.626, p = 0.055). The follow-up pairwise comparisons confirmed that the base radius increased significantly with age (p < 0.05) between any two age-group pairs except between 12-mo and 18-mo. Similarly, the minimal pupil radius increased with age (p < 0.05) between any two age-group pairs except between 12- and 18-mo and 6- and 12-mo. The relative constriction showed an overall increasing trend with age. However, the difference reached significance only between 6- and 12-mo, and between 6-mo and 24-mo. The decreasing tend in latency was significant (p < 0.05) between 6- and 24-mo, 12- and 24-mo, and between 18- and 24-mo. The pairwise comparison revealed that the decreasing trend in response time was significant between 12- and 18-mo, and between 12- and 24-mo.

## Discussion

**Figure 4:**
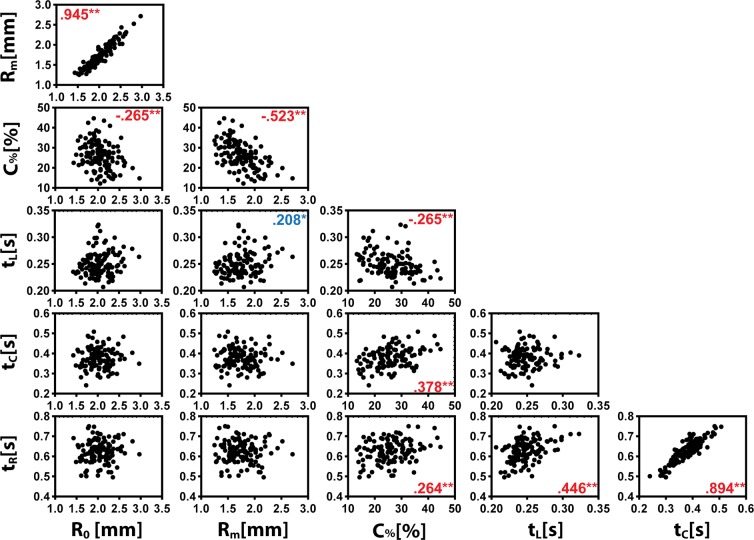
Correlations among the six PLR parameters. The numbers in plots indicate Pearson’s correlation coefficients. **Correlation is significant at the 0.01 level (2-tailed). * Correlation is significant at the 0.05 level (2-tailed).

This study revealed significant age trends in the pupil size, constriction, latency, and response time in 6- to 24-month children. The trend seen in the base pupil radius was consistent with previous reports that pupil size increased from birth until teenage years in typically developing children^17,18^. The observation that male children had slightly bigger pupil size was also in agreement with previous studies^17^. A close examination of the correlations among the six PLR parameters (Fig. 4) indicated that base pupil radius and minimal pupil radius were highly correlated (Pearson correlation r = 0.945). Therefore, the similar effects of age, sex, and group on base radius and minimal radius can be expected.

The increase in relative constriction with age, in particular from 6-mo to 12-mo and to 24-mo, appeared to be consistent with a previously observed trend in 2-year to 3-year old children^12^. The age trends in base and minimal pupil radii were opposite to that of the constriction. Such opposite trends cannot be simply explained based on correlation. As shown in Fig. 4, the relative constriction only had a week negative correlation with base radius (r = −0.265) and a moderate negative correlation with minimal radius (r = 0.523).

The observation of latency decreasing with age was consistent with previous results reported in 2- to 6-year and 6- to 18-year old children of typical development^11,12^. Taken together, these data suggested that PLR latency decreases from 6 months old until 9~10 years old in the typically developing children. A similar decreasing trend was observed in the PLR response time, although the response time had only moderate correlation with latency (Pearson correlation r = 0.446). The PLR constriction time was not correlated with latency and showed no significant age trend.

The observation that base pupil size was larger in the HR group than in the LR group appeared similar to the difference previously reported between 2-year old children with ASD and those of typical development^12^. The observation of a smaller relative constriction in the HR group was similar to that observed in older children affected by ASD using a desktop PLR device^10,11^. Following previous studies^11,23^, the relative or normalized constriction was used as a way to compensate the variations caused by different baseline pupil sizes. Interestingly, a careful examination indicated such a difference in relative constriction was due to the larger resting pupil radius which was the denominator in calculating the relative constriction C_%_ = (R_o_^2^ − R_m_^2^)/R_o_^2^. No significant group effect was observed when the simple pupil size change R_o_ − R_m_ was analyzed using the LMM analysis. Nevertheless, this observation was inconsistent with a previous study by Nyström *et al*. who reported that PLR constriction was larger in 9–10-month-old with high risk of ASD^16,23^. Such inconsistency may be attributed to different methodology and testing conditions used. There were significant variations in the room lighting conditions and optical stimulations among previously reported studies due to different testing systems used. Changes in light adaptation and optical stimulation can greatly affect the PLR response and may lead to altered age trends^22^.

The PLR differences observed between HR and LR children are similar to those reported in older children between those affected by autism and those of typical development. Presumably, only very few in the HR group may be eventually diagnosed with an ASD. Such observation could be attributed to genetic or possible environmental factors; but further studies are necessary to understand this. The lack of a strong correlation between based radius, relative constriction, latency, and constriction time may suggest these parameters are modulated under different neurological mechanisms. A bigger pupil size and a smaller constriction may be consistent under a stronger sympathetic modulation^8^. The constriction speed is controlled by iris muscle contraction and thus is more under the influence of parasympathetic modulation. On the other hand, the latency represents essentially the neural signal transduction and processing speed, which could be affected by synaptic function, white matter maturation, or network connectivity, all implicated in ASD^30–33^.

Two children in the HR groups received diagnoses at the end of this study: one was diagnosed with ASD and the other with global developmental delay. The one diagnosed with ASD had PDDST-II score of 7 at both 12- and 18-mo; whereas the other had a score of 4 at 12-mo and 6 at 18-mo. When examining the PLR results from these two participants against the entire data set, no obvious distinct patterns were observed for the child diagnosed with global developmental delay. However, the one who received an ASD diagnosis showed some interesting patterns (Fig. 3). First, this participant had a latency of 206.6 ms at 12-mo, the smallest among all participants, which increased to 233.3 ms at 18-mo. Meanwhile, the constriction time decreased greatly from 455.6 ms at 12-mo to 284.7 ms at 18-mo, which was the largest reduction among all participants. It is interesting to note that the observation of a small PLR latency at 12-mo in the one diagnosed with an ASD appeared to be consistent with previous speculation that ASD may be associated with shortened latency in infants, but longer latency in older children^11,12,23^. Such age dependent difference suggested the possible use of PLR as an indicator of atypical developmental trajectory in children.

In summary, we conducted a longitudinal study of PLR behaviors in 6-mo to 24-mo children with and without high risk of developing ASD. The results indicated significant age trends in base pupil radius, minimal pupil size, and latency. Specifically, this study showed that the base and minimal pupil size increased with age significantly, while the latency decreased significantly during this period. Furthermore, atypical PLR parameters seen in previous studies of older children with ASD were also observed in younger children age 6–24-months. We have found that the pupils of the children with higher risk of ASD were, on average, larger at both resting state and the time of maximal constriction. The one participant who was diagnosed with ASD at the end of the study showed some distinct patterns in PLR latency and constriction speed. Additional studies in a large population are necessary to further evaluate these observations. In future studies, it will be valuable to also assess the effect of developmental age in addition to chronological age. More advanced data analysis methodology such as Bayesian factor analysis may also be employed to explore further the interactions among different factors.

## Methods

### Participants

Forty-two children participated in this study. All participants were recruited through the Thompson Center for Autism and Neurodevelopmental Disorders at the University of Missouri (MU). Twenty-three participants made up the high-risk group (HR), which consists of children who have at least one sibling diagnosed with ASD. The low-risk (LR) group had 19 children who have no family history of autism or other neurodevelopmental disorders. This study was approved by the Institutional Review Board (IRB) of the University of Missouri. All methods were performed in accordance with the relevant IRB guidelines and regulations. Written informed consents were obtained from the parents/guardians prior to the PLR test.

Table 2 shows the number of participants at each of the four nominal testing ages of 6-, 12-, 18-, and 24-month. The actual age distributions were also shown in the table. The test data from one girl at 6-month and one girl at 12-month, both from the HR group, were not successful because they either could not look at the screen or their excessive movement did not allow clear pupil images to be recorded. The final dataset consists of PLR measures from nine children (6 in HR and 3 in LR) who successfully completed test at all four ages, 24 children (9 in HR and 15 in LR) who successfully completed tests at three ages, and nine children (8 in HR and 1 in LR) who only completed tests at two or one age.

**Table 2:** Number of participants and their age distributions at the four nominal testing ages (F: female; M: male).

		6-mo	12-mo	18-mo	24-mo
Low-risk	Number	2 F/3 M	9 F/10 M	9 F/10 M	8 F/8 M
	Actual age	6.0 ± 0.7 mo	11.3 ± 1.3 mo	17.4 ± 0.5 mo	23.7 ± 0.5
High-risk	Number	5 F/6 M	9 F/13 M	8 F/12 M	6 F/8 M mo
	Actual age	6.3 ± 0.8 mo	12.2 ± 1.1 mo	17.6 ± 0.6 mo	23.4 ± 0.5 mo

One participant in the HR group reported vision problems due to Usher syndrome^34^. This child’s PLR results were included in the overall data analysis because they did not show any obvious differences from other children’s data in the group. All other participants reported neither vision problems nor any family history of eye disorders. Participants were requested to withhold medications 48 hours prior to the test, unless it was necessary. Two subjects received vaccinations within 24 hours of testing of their 6- and 12-month tests. Three subjects reported to have taken antibiotics before their tests (two at 6-months and one at 24-months). Children in the LR group are typically developing during the study period based on the family’s report of their most recent well-baby checkups. In addition, the Pervasive Developmental Disorders Screening Test-II (PDDST-II, Pearson Clinical Assessment)^29^ scores were recorded as a simple screening for neurodevelopmental disorders in the participants before 24 month of age. By the end of this study, two HR participants received diagnoses at the MU Thompson Center: one with ASD and one with non-ASD global development delay. Another participant was reported to have minor speech delay. No developmental problems were reported by the parents of other participants on their recent well-baby checkups.

### Test procedure

All participants were tested using a remote PLR (rPLR) instrument. The details of the system and testing arrangement have been described in detail previously^27^. The rPLR utilizes a tracking system to follow the position of the subject’s right eye. The data of the eye’s position is then used to focus and change the direction of the PLR imaging camera to the subject’s right pupil. This design enables PLR measurements in children without the need of a strict physical restraint. The PLR tests were conducted in a bright room with illuminance level measured at ~ 120 lux. The participants were placed in front of the rPLR system in a car seat or seated on a parent’s lap. Cartoon videos were shown on a projection screen on the wall ~ 200 cm from the participants to maintain their attention during the test. The videos that displayed on the projection screen had a size of 81.3 cm × 55.9 cm (width × height). PLR was elicited by flashing the projection screen using a ceiling-mount green LED (530 nm wavelength). We focused on studying the transient constriction phase of the PLR induced by a brief 100 ms flash as in previous studies^10–12^. The transient PLR responses are mainly mediated by the cone and rod photoreceptors under photopic conditions^35–37^. The stimulation wavelength used in this study is approximately midway between the wavelengths at the peak sensitivities (V-lambda) of rods and cones. The stimulus light intensity at the position of the eye was calibrated as 4.1 μW/cm^2^ (13.0 log photons cm^−2^ s^−1^). The rPLR system used 850-nm near infrared (NIR) LED array to illuminate the subject’s right pupil for imaging.

Each PLR test lasted less than 10 minutes with about 20–25 PLR trials recorded from the participants as they watched the cartoons. There was a minimum of a 20 s interval between two consecutive trials. To begin with, the participant was given a few minutes to get comfortable and acclimate to the testing room environment. Within each PLR trial, the pupil images were recorded for 2 s starting 0.25 s prior to the 100 ms optical stimulation.

**Figure 5:**
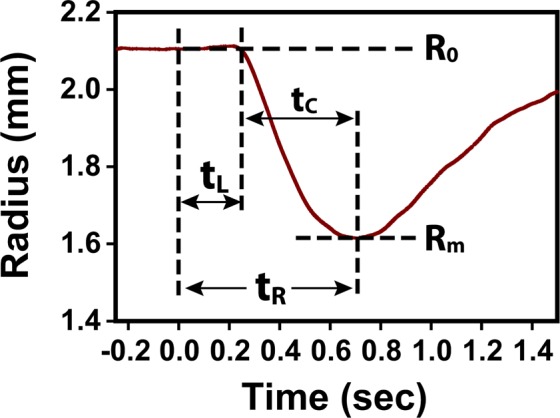
An illustration of the quantitative PLR parameters extracted from a measured pupilogram.

The PLR data recorded from all trials in each test were processed off-line to create the pupilogram curve to quantify the change of pupil size with time. Similar to previous studies^10,11^, the following six PLR parameters were extracted from the resulting pupilograms to characterize the constriction phase of the pupil responses (Fig. 5).

1. The baseline pupil radius R_o_ was calculated as the average pupil radius prior to the light stimulation onset.
2. The minimal pupil radius R_m_ was calculated as the smallest pupil radius during constriction.
3. The relative constriction C_%_ of the pupil was calculated as C_%_ = (R_o_^2^ − R_m_^2^)/R_o_^2^. This PLR measure normalized changes in pupil area against the baseline pupil area.
4. The PLR latency t_L_ was calculated as the time interval between the beginning of the stimulation and the onset of the pupillary constriction. The constriction onset was determined as the first deflection data point when pupil started to constrict consistently.
5. The constriction time t_C_ was calculated as the time interval between the onset of the constriction and the minimum pupil radius size.
6. The response time t_R_ was calculated as the time interval between the stimulation onset and when pupil reaches the minimal size, which was equivalent to t_L_ + t_C_.

Multiple PLR trials were acquired during a single test. All PLR trials that could not be used to construct the pupilogram were discarded. These failed trials were generally caused by excessive eye/head movement. The PLR parameters were calculated from all remaining successful PLR trials. Not all PLR parameters could be obtained from all trials due to eye closing, movement, or blinking within the 2 s acquisition window. For each PLR parameter, trial results were considered as outliers if the values were more than 3 times of the Scaled Median Absolute Deviation (MAD) away from the median of all successful trial results measured in the same test. After removing all outliers, the mean PLR parameters from all remaining trials were calculated and used in the final data analysis. The average and standard variation of numbers of good PLR trails for each parameter are shown below in Table 3. The majority of tests yielded at least five good PLR trials that were included in the final data analysis.

**Table 3:** The distributions of number of outliers removed and the remaining good PLR trials for each PLR parameter.

		R_o_	R_m_	C_%_	t_L_	t_R_	t_C_
Outliers	mean ± std	0.3 ± 0.7	0.3 ± 0.7	0.4 ± 0.7	0.3 ± 0.6	0.4 ± 0.7	0.3 ± 0.7
	% ≤ 1	93.7%	92.1%	89.7%	92.1%	88.9%	91.3%
Good trials	mean ± std	16.5 ± 5.3	12.8 ± 6.0	12.4 ± 5.9	12.2 ± 5.6	12.1 ± 5.9	11.0 ± 5.5
	% ≥ 5	99.2%	89.7%	88.1%	89.7%	84.1%	82.5%

### Statistical analysis

A linear mixed-effects model (LMM) was applied with maximum likelihood method to determine the main effects of participant group (HR or LR), age, and sex on PLR parameters. A random intercept model was applied. The effect of age as represented in four age groups (6-, 12-, 18-, and 24-month) was treated as repeated measure with “Scaled Identity” as the repeated covariance type. Neither the group × age interaction, nor the sex × age interaction, nor the group × sex interaction was found significant during the model selection process. Therefore, no interaction term was included in the final LMM analysis. Follow-up pairwise comparison with Bonferroni confidence interval adjustment was used to compare mean PLR parameters between different groups. Alpha was set at 0.05 for all statistical tests. All statistical analysis was conducted in IBM SPSS Statistics V25.

## Supplementary information

Supplementary Figure S1.

## Data Availability

The datasets generated and/or analyzed during the current study are available from the corresponding author on reasonable request.
