# Supplementary material for: A longitudinal study of pupillary light reflex in 6- to 24-month children
Source: Sci Rep. 2020 Jan 27;10:1205. doi: 10.1038/s41598-020-58254-6 (PMC6985190; doi:10.1038/s41598-020-58254-6)
Supplement: Supplementary file 1 — Supplementary Figure S1. [file 41598_2020_58254_MOESM1_ESM.pdf]

A longitudinal study of pupillary light reflex in 6- to 24-month children

Clare Kercher[1], Leila Azinfar[1], Dinalankara M. R. Dinalankara[1,2], T. Nicole Takahashi[3], Judith H. Miles[3], and Gang Yao[1*]

Authors' institutional affiliations:

[1] Department of Biomedical, Biological & Chemical Engineering, University of Missouri, Columbia, MO 65211, USA

[2] Department of Computer Engineering, University of Sri Jayewardenepura, Nugegoda, Sri Lanka

[3] Thompson Center for Autism and Neurodevelopmental Disorders, University of Missouri, Columbia, MO 65211, USA

Corresponding author: Gang Yao, University of Missouri, 249 AG ENG Bldg, Columbia, MO 65211-5200; 573-884-7529; YaoG@missouri.edu

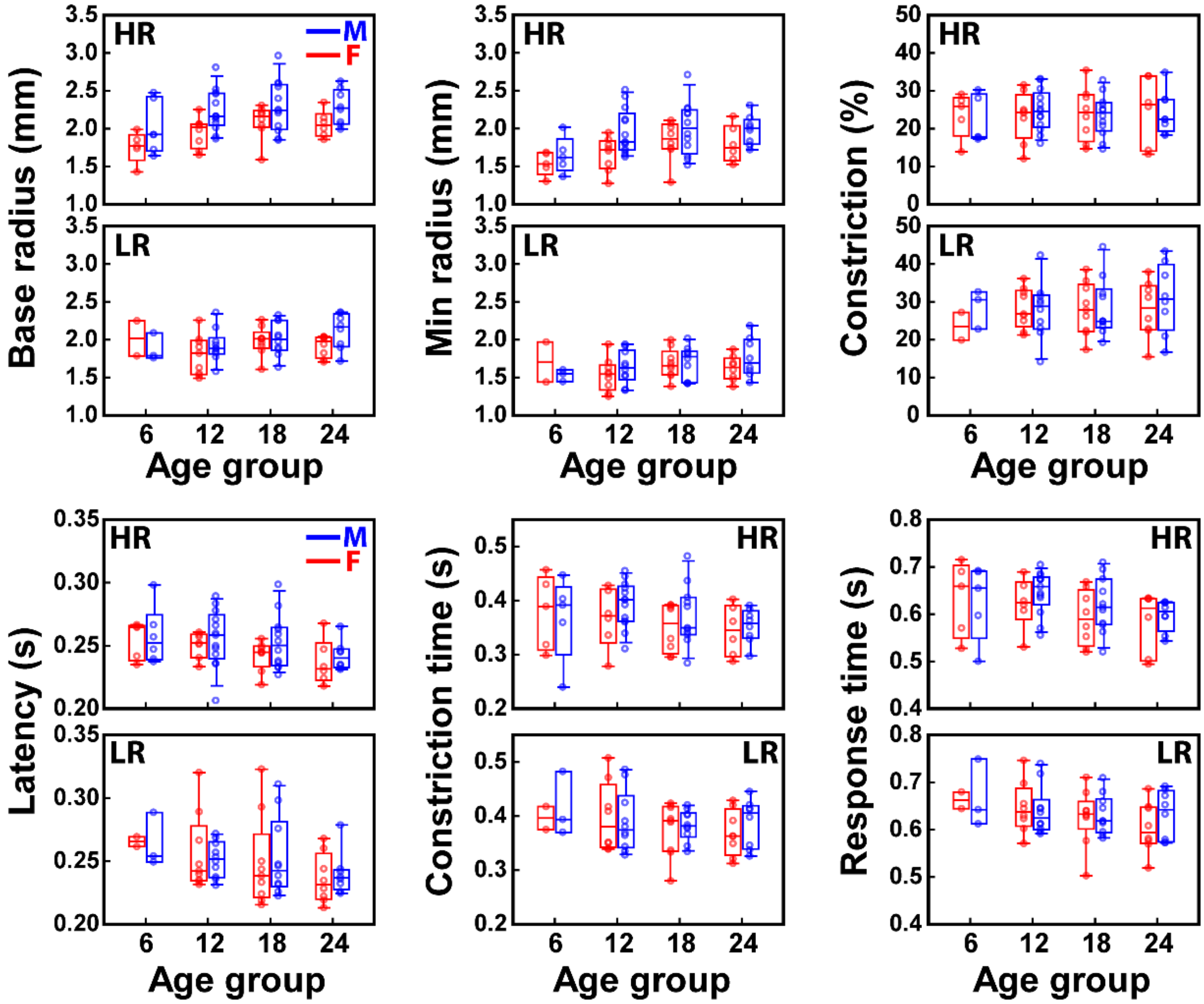

Supplementary Figure S1. The Box plots of extracted PLR parameters (base radius, minimal radius, relative constriction, latency, constriction time, and response time) in all participants at different age groups. The data were separated into the high-risk (HR) and low-risk (LR) groups, and males (M) and females (F) in each group. The box boundaries indicate the 25th and 75th percentile; the line within the box represents the median; and error bars indicate the 90th and 10th percentiles.
